# Wind Pollination of Apple Flowers Under Insect Exclusion Nets Questions the Insect-Dependent Pollination Model of Modern Apple Plantations

**DOI:** 10.3390/plants14081196

**Published:** 2025-04-11

**Authors:** Mokhles Elsysy, Aziz Ebrahimi, Todd Einhorn

**Affiliations:** 1 College of Agriculture and Natural Resources, Michigan State University, Horticulture 1066 Bouge ST, East Lansing, MI 48824, USA; 2 Department of Pomology, College of Agriculture, Assiut University, Assiut 71515, Egypt; 3 Department of Forestry and Natural Resources, College of Agriculture, Purdue University, West Lafayette, IN 47907, USA; aebrahi@purdue.edu

**Keywords:** crop load, fruit set, KASP SNP markers, fertilization, floral biology

## Abstract

Pollination is essential for producing temperate-zone tree fruits like apples (*Malus* × *domestica*). While traditionally considered insect-dependent, this view may result from orchard designs tailored to European honeybees. Previous research showed that low-seed apples could develop in insect exclusion nets, suggesting wind as an alternative pollinator. This study investigated the paternal origin of seeds and fruit set under nets compared to open canopies. Netted canopies of ‘Gala’, Fuji’, and ‘Honeycrisp’ set commercial fruit numbers without manual thinning. To determine the parental source of seeds, genotyping was performed using 16 SNP markers tailored for distinguishing apple cultivars, with primer design and genotyping conducted via the KASP™ system. Results showed significant genetic overlap between seeds from netted and non-netted fruits and nearby pollinizers, ruling out self-pollination. Netted canopies retained fruits with similar or fewer seeds compared to abscised fruits in open canopies, indicating fruit set depends on the population’s seed content rather than individual fruit seed count. These findings supporting the hypothesis that apple trees are adapted to utilize both wind and insect pollination. While wind pollination offers a sustainable approach, it requires adjustments in orchard design to ensure sufficient pollen transfer for reliable fertilization and yield.

## 1. Introduction

Flowers of domesticated apple (*Malus* × *domestica Borkh.)* are perfect and complete. Apple is regarded as a self-infertile member of the Rosaceae family whose outcrossing is facilitated by the gametophytic self-incompatibility (GSI) system requiring pollen donors to possess at least one different S allele than the pistil to facilitate fertilization [1]. Pollination is a necessary step in apple fruit development, except for seedless cultivars (e.g., Spencer Seedless’, Wellington Bloomless’, and Rae Ime’) whose fruit originate parthenocarpically [2]. In rare cases, self-compatible and cross-incompatible cultivars exist, albeit they are few [3]; the latter is limited to genetically similar varieties such as Cortland, Macoun, and McIntosh, and sports of a cultivar [4]. Self-pollination studies have demonstrated varying levels of self-fertility, ranging from ~2% in ‘Fuji’ and ‘Golden Delicious’ to 8% and 12% for ‘Elstar’ and ‘Idared’, respectively [5].

Pollination of most tree fruit species, including apple, is considered insect-dependent (Entomophilous), purportedly due to the relative mass of tree fruit pollen [6], in contrast with anemophilous species such as nut trees that depend on wind for pollen dispersal. The primary apple pollinators comprise species of bees and hoverflies [4,7]; the former are highly effective pollinators of apple flowers [8] and, thus, are typically introduced to apple plantations as managed hives of European honeybees (*Apis mellifera*) to ensure adequate fruit set for commercial yield [4]. An important agronomic consequence of current apple pollination practices is the ‘oversetting’ of fruit, necessitating the additional expense and often unpredictable practice of chemical fruitlet thinning [9]. Several native insect species such as mason bees (e.g., *Osmia* spp.), mining bees (e.g., *Andrena* spp.), and bumblebees (*Bombus* spp.) can also effectively pollinate apple [10]. The diversity and density of these insects varies considerably among and within geographic regions depending on habitat and cultural practices [11]. Sustainability of managed hives is threatened by pollinator decline [12] in addition to several anthropogenic factors, namely climate change, but also insecticide use and habitat loss [13,14].

Wind pollination of apple trees was challenged and dismissed for commercial production [7]. The common assertion that wind-disseminated pollen loads were insufficient to produce consistent commercial crops has been maintained [15]. Air samples collected within apple orchards, however, contained apple pollen [16,17], as did stigmatic surfaces [18]. Distances of apple pollen transported by wind range from 5–6 m [19] to 20 m [20]. Similar results were obtained in pear orchards (*Pyrus communis* L.) [21,22,23]. Recovery of TNR 31–35 hybrid pollen, having a red pigmented phenotype, was ~69% when <10 m from the nearest pollen dispenser and 91% when the distance was increased to <60 m [20]. Similarly, [24] Soejima, 2005 captured 75% of crabapple pollen within 10 m of its release and 95% within 60 m. Pollination from GM ‘Gala’ carrying the GUS marker gene (at heterozygous state) declined over a 30 m distance from the source (i.e., 0.24% of pollen recovered) but was shown to travel 137 m as determined via the genetic characterization of transgenic seedlings over that distance [25]. Similar results were obtained by Kron et al., 2001 [26] in two different orchards, where approximately 44% and 80% of seedlings within 14.5 m of the donor were transgenic, and 20% and 1% of seedlings were transgenic at 73.5 m and 86 m, respectively, in both orchards. Outcrossing in natural populations of crabapples is also common within 50 m from, and up to 300 to 500 m from, the pollen donor [27], and, infrequently, over much greater distances of 10.7 km [20]. Collectively, ample evidence exists for wind dissemination of pollen to fertilize apple flowers; the central question is whether pollen loads and, ultimately, fertilization rates are sufficient to set full crops in apple plantations. It must also be acknowledged that tree density and planting arrangement, including ratios of main cultivars to pollinizers, contribute to pollination efficacy [28,29], and their design has not been considered to facilitate wind pollination.

The use of netting has become widespread in commercial apple plantations to eliminate specific insect pests (namely, codling moth) and protect fruit from hail damage [30]. We previously demonstrated adequate fruit set and production of apple when enclosing canopies at relatively low percentages of open bloom [9,31], a strategy intended to reduce or eliminate chemical thinning by preventing bees from pollinating excess flowers. One notable observation from that work was the ability of some cultivars to set a full crop of low-seed-content fruit when nets were enclosed prior to any flowers reaching anthesis. Without insects to disseminate pollen, we hypothesized that the origin of the pollen was external to the nets, having arrived via the wind, but self-fertility could not be ruled out [9,31]. Ref. [32] Langridge & Jekins observed a reduction in wind-disseminated pollen through bee-proof enclosures (i.e., 20% less airborne pollen than non-enclosed canopies), though others have demonstrated sufficient transfer of apple pollen into netted canopies [33,34]. The current study aimed to investigate the role of wind in apple pollination on fertilization rates by characterizing seed number per fruit and demonstrating its influence on abscission of developing fruitlets, as well as to confirm self- or cross-pollination of flowers via the KASP™ system. We hypothesized that fruit set under nets was from cross-pollination and that apples could produce sufficient crop loads (between 5 and 8 fruit per cm^2^ of TCSA), but not superfluous fruit set when wind was the only available pollinator. As an agronomic tool, the use of nets in combination with altered planting designs might provide better pollination services by increasing economic and environmental sustainability.

## 2. Results

Fruit set: Netting trees at ‘pink’ stage (i.e., pink tightly closed petals with 0% of flowers open at the time of net enclosure) significantly reduced fruit set compared to open-canopy, non-netted trees in both years for all cultivars. Although the percent reduction varied from 20% to 64% among cultivars and between years, the percent reduction in fruit set of netted ‘Honeycrisp’ compared to open-canopy trees was highest compared to other cultivars, with 64% and 50% in 2021 and 2022, respectively. An episodic spring frost in 2021 led to relatively high percentages of pistil mortality of ‘Fuji’ flowers compared to the other cultivars, regardless of treatment; irrespective, netted canopies had 35% and 40% reduced fruit set compared to open, non-netted canopies in 2021 and 2022, respectively (Table 1). Fruit set within ‘Gala’ canopies was the least affected by nets, being reduced by ~30% over the two years compared to controls.

Seed content: Netted canopies of all cultivars produced seeded fruit in both years (Table 2). The average number of developed seeds per fruit from netted canopies of ‘Gala’, Fuji’, and ‘Honeycrisp’ was 1.8, 1.2, and 4, and 5.2, 5.1, and 5.4 for 2021 and 2022, respectively. A significantly higher number of developed seeds per fruit was observed from non-netted canopies in all cases except ‘Honeycrisp’ 2022 and ‘Fuji’ 2021 (Table 2). The number of developed seeds per abscised fruit from non-netted canopies was similar to the number of developed seeds per retained fruit from netted canopies, except ‘Gala’ (2022) and ‘Honeycrisp’ (2021); in these two cases, retained fruit of netted trees had lower developed seed content than abscised fruit of non-netted canopies. The effect of seed number per fruit on fruit abscission was highly significant, irrespective of treatment or cultivar. With the exception of Gala 2022, the seed number in retained fruit was approximately 50% higher than that in abscised fruit (Table 2).

**Figure 1:**
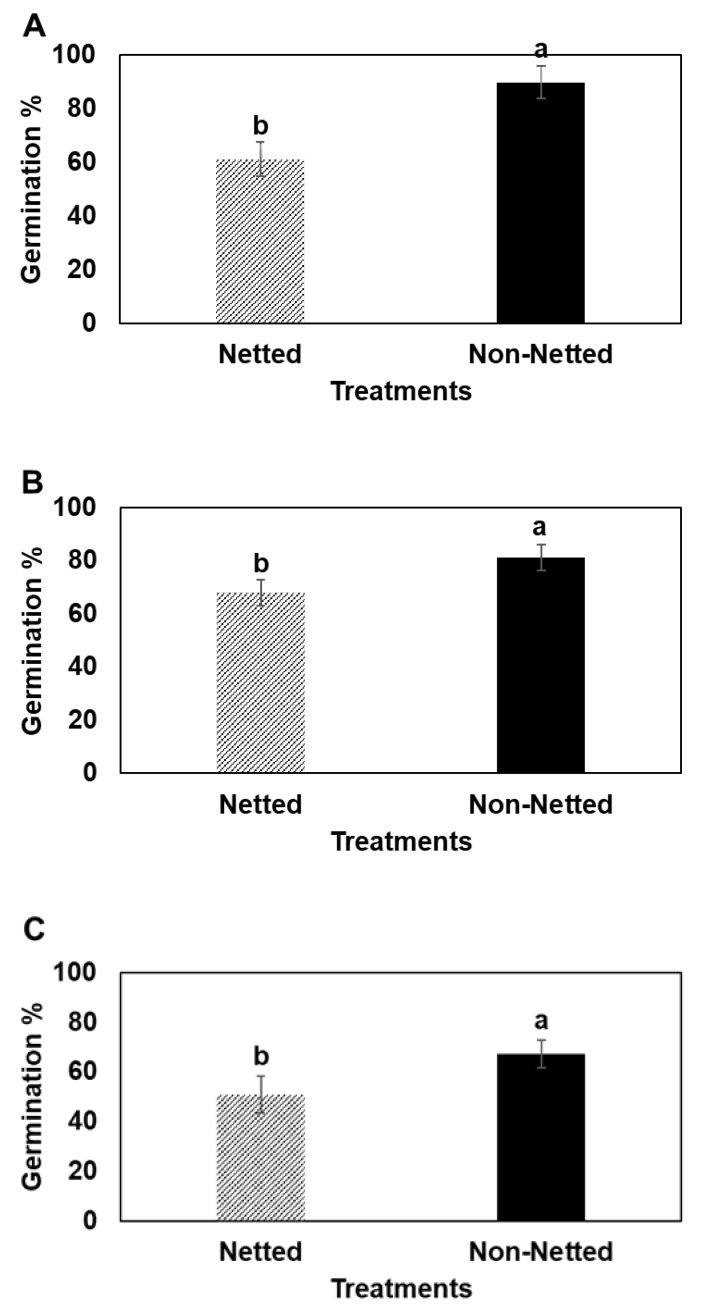
Germination percentage of seeds produced in netted and non-netted trees in spring 2021 of (**A**) ‘Gala’, (**B**) ‘Honeycrisp’, and (**C**) ‘Fuji’. Data are means of four or five single-tree replicates, and each tree is a mean of ~25 fruit. Bars are ±1 SE. Mean separation among treatments by Tukey HSD (*p* < 0.05), whereby means associated with different letters are significantly different.

Germination: Germination rates of seeds were significantly higher from fruit of open, non-netted canopies than netted canopies in all cultivars (Figure 1).

**Figure 2:**
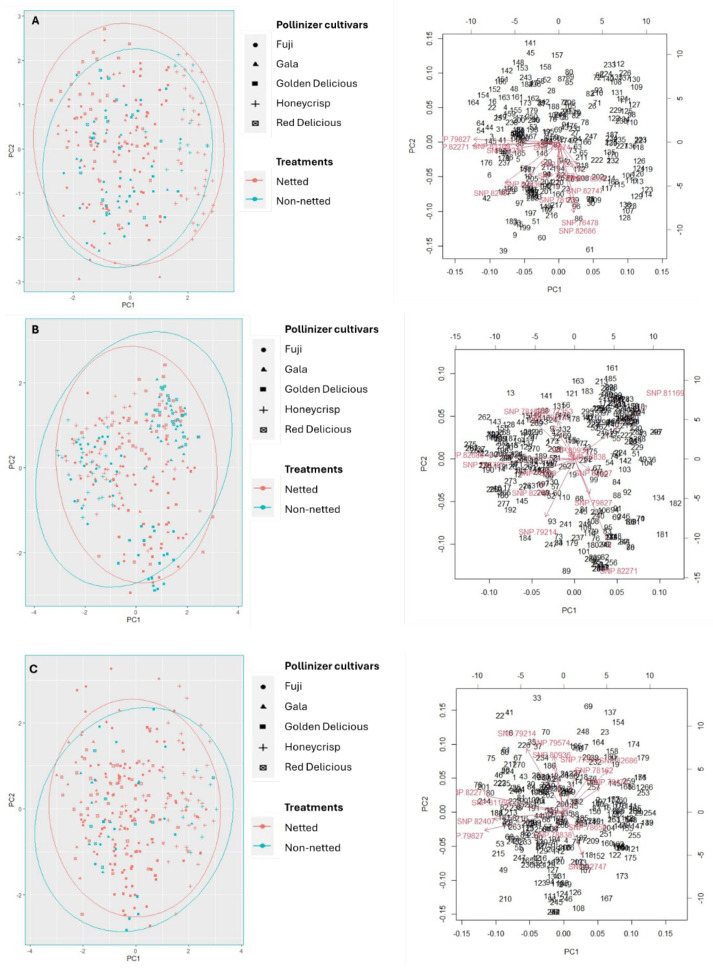
PC score plots of SNP calling for KASP markers (82271, 77353, 82686, 75890, 82747, 78162, 78658, 78838, 79214, 79574, 79827, 80054, 82407, 80936, 81169, and 76478). Sample position is determined by intensity of signal detected from fluorochromes bound to allele-specific primers. Samples of similar signal with each of the possible pollinizer cultivars were assigned a value of 0, 0.5, or 1 based on X and Y values. Data are collected from 5 trees of each cultivar and 10 to 15 fruit of each tree. (**A**) Gala, (**B**) Honeycrisp, and (**C**) Fuji.

Genotyping analyses: A comparative analysis using KASP markers among seedlings germinated from seeds of fruit from netted and open canopies of ‘Gala’, Fuji’, and ‘Honeycrisp’ and leaves of potential pollinizers in close proximity to treatment trees indicated no significant variance in paternal origin between the netted and non-netted canopies (Figure 2). Thus, the likelihood of adjacent cultivars serving as potential pollinizers was found to be comparable for both netted and non-netted environments within ‘Gala’, ‘Honeycrisp’, and ‘Fuji’, and the data do not support self-fertility as the mechanism for pollination. Among the 16 KASP markers analyzed, three markers indicated no discernible differences between the genotypes of the seedlings and those of the pollinizer cultivars. Moreover, the genetic distribution of the remaining 13 markers showed a significant overlap between the netted and non-netted canopies, with over 90% of the samples clustering within a range of 2 and −2 along the principal components PC1 and PC2.

Fruiting efficiency indices: Crop loads of open-canopy, non-netted trees were significantly higher than those of netted trees in all years and cultivars (not withstanding ‘Fuji’ in 2021), resulting in excessive fruit set relative to optimum crop load indices for the individual varieties by an average of one fruit/cluster (Table 1). Low crop loads of ‘Fuji’ trees in 2021, regardless of netting treatment, resulted from a combination of freeze-induced mortality and trees being in an ‘off’ year due to the biennial nature of ‘Fuji’. Crop loads of netted canopies were at or near (±5%) commercial crop load indices [9,31] for the individual varieties in 4 of 5 scenarios, not including the freeze-affected Fuji.

## 3. Discussion

The role of wind in apple pollination has received little attention since the 1960s when it was considered impractical for producing sufficient commercial apple yields [7], a sensible conclusion for orchards of that period given their voluminous, three-dimensional canopies and low planting densities. Apparent bee preference for travel along rows, as opposed to across rows, supported this assertion. Several reports of that era, however, documented fertilization of netted apple and pear trees via wind-dispersed pollen [21,22,23]; planting distance between single-tree pollinizers and main cultivars (ratios of ~0.12) of those orchards was designed with consideration to honeybee flight habits and efficacy for pollination, not wind [6,29,35]. Presently, honeybees remain the key pollinator of apples [4], although canopy architecture and planting designs have changed considerably. Modern apple plantations comprise narrow continuous canopies (i.e., fruiting walls) whereby pollen dispersal has been recorded at distances of 86 m across rows and significantly higher than along rows, although most pollen deposited within 14.5 m of the source [25]. In the orchard of the present study, the farthest row within a five-row replicate of any cultivar (i.e., the center row) from an entire row of pollinizer trees was a mere 10 m, undoubtedly aiding the success of wind pollination. A complete lack of fruit set of caged apple limbs in an orchard without pollinizer trees was increased to ~50% when pollen was mechanically applied using forced advection with an electrostatic sprayer [36], corroborating our results of wind efficacy given the proximity of pollen donors.

Native pollinators contribute significantly to apple fruit set, regardless of honeybee presence in the orchard [37], although this varies among regions. Agronomic plantations of apple void of managed honeybees have been shown to produce equivalent rates of fruit set as those with honeybees [38], although the relative contribution of wind and alternative insect pollinators when honeybees were present was not assessed. Nevertheless, wind-dispersed apple pollen traveled distances of 20 m, depending on wind direction, and transported through porous enclosures to pollinate flowers [20]. Evolutionary studies in some angiosperm species suggest a combination of insect and wind pollination to be more common and representative of a stable or transitional state from insect pollination [39]. These principles no doubt have application to agronomic systems comprising tree fruit species but have not, so far, received adequate attention. Since native and introduced (i.e., honeybee) insects were excluded by the tiny orifices of the net system in the current study, any pollination would have required other processes.

The current study demonstrates that wind was not only efficient for pollinating pistils of apple flowers when bees were excluded but, in fact, set optimum crop loads, as evident by crop load levels in all but Honeycrisp in 2021 (excluding the freeze-induced mortality of ‘Fuji’ flowers in 2021; Table 1). ‘Royal Gala’ and ‘Golden Delicious’ trees trained in modern canopy configurations set commercial yields under nets ~(0.8 and 1 kg·cm^−2^, respectively) [40], as did ‘Golden Delicious’ and ‘Gold Rush’ [41], although nets were not applied prior to bloom in those studies. We previously demonstrated effective pollination and fertilization of apple flowers enclosed in insect exclusion nets before anthesis but did not attempt to elucidate the pollen source [9,31]. Subsequently, we hypothesized that enclosed flowers may have been ‘selfed’ given the combined results of reduced fruit set and low seed content compared with non-netted controls; the plausibility of this is supported by accounts of self-fertility, albeit to a limited extent, in certain apple varieties [5]. SNP-KASP marker analysis, however, ruled out self-pollination within nets in the present study (Figure 2). These data compel us to accept that alternative pollen sources were disseminated by wind.

**Figure 3:**
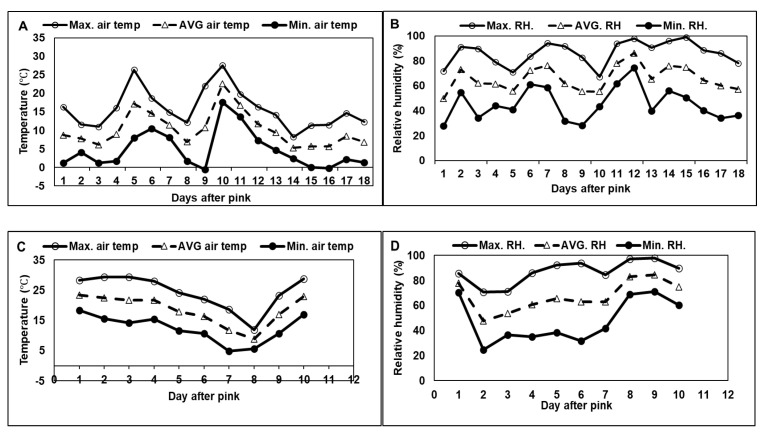
Daily minimum, maximum, and average air temperature (**A**,**B**) and relative humidity (**C**,**D**) for 2021 and 2022 respectively.

**Figure 4:**
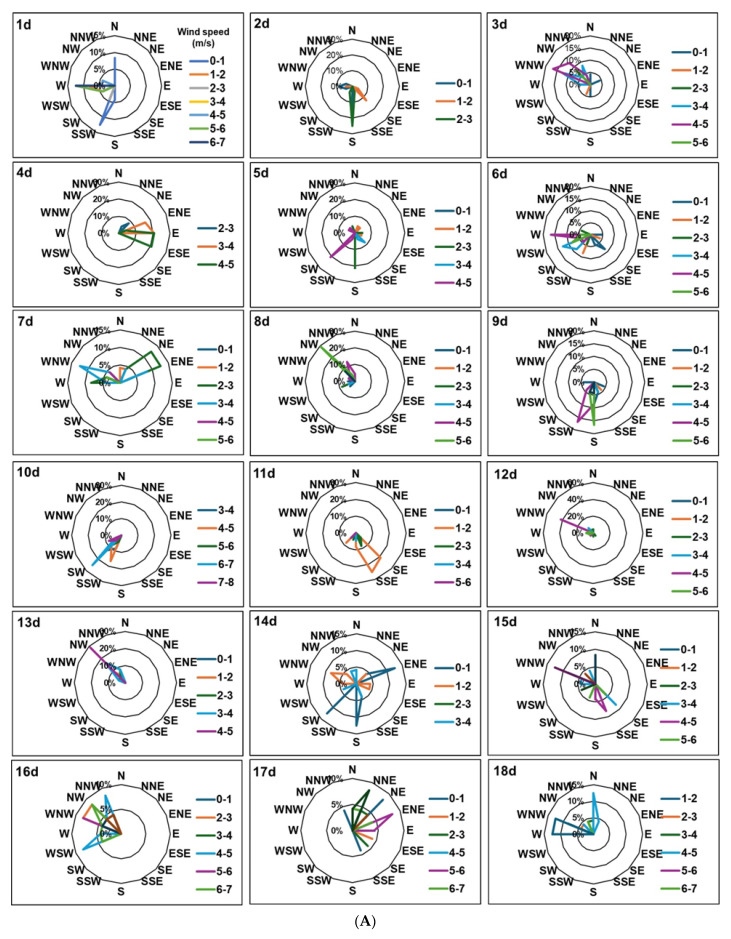

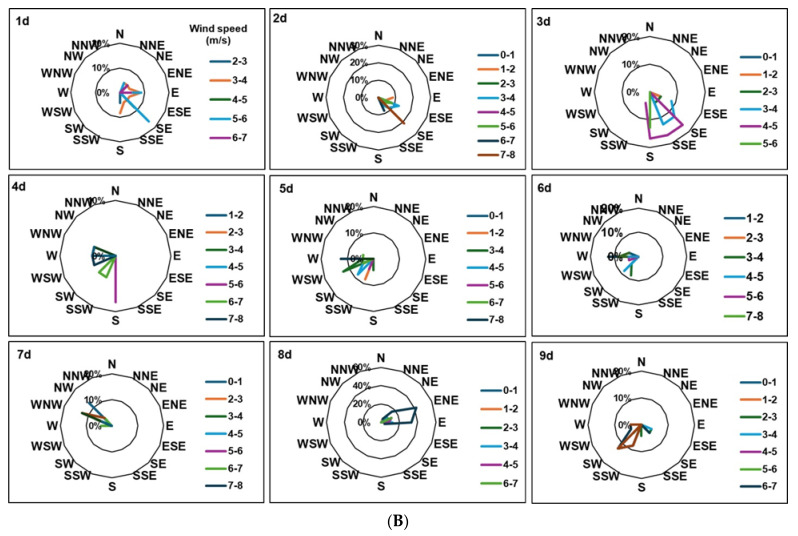
Hourly wind speed and direction in Clarksville, Michigan (42°50′27″ N 85°14′36″ W). From 23 April 2021, pink stage (0% KB) to 10 May 2021, five days post full bloom (peta fall) in 2021 (**A**) and 2022 (**B**), respectively.

Pollination efficiency partly depends on the kinetics of pollen tube growth and this growth rate is widely established to be dependent, positively, on temperature [42,43]. Based on these studies, temperature measured in 2022 would have facilitated pollen tube growth to the base of the style within 24–48 h (Figure 3). These high temperatures also substantially truncated the 2022 bloom period compared to 2021. In addition, higher wind speed during the 2022 bloom period would have improved pollen dispersal and distance, potentially increasing fruit set rates and seed content compared to 2021, as observed. Wind speed during the nine-day bloom period of 2022 was on average 2 m·s^−1^ higher than the bloom period of 2021 and wind direction was markedly more disparate relative to 2021 (Figure 4). Adequate pollen to achieve full crop loads was not likely deficient within netted canopies in 2021. The lower pollination efficiency of wind (versus bees), however, places greater importance on climatic conditions, namely, temperature, but also wind, when combined with netting.

Seed content significantly affected fruit abscission in netted and non-netted trees alike, but notably, retained fruit of netted trees had similar or lower seed content than abscised fruit from non-netted trees. That fruit set is a relative condition based on a given population’s seed content implies that lower rates of fertilization under nets facilitated the set of fruit that would have otherwise abscised from non-netted trees. Theories explaining fruitlet abscission generally agree that reduced polar auxin transport enhances fruitlet sensitivity to ethylene, resulting in abscission-zone formation [44]. The underlying factors that contribute to reduced polar auxin transport include carbohydrate stress, resulting in ethylene biosynthesis and embryo death of non-developed seeds [45] and the dominance of earlier developing ovaries and fruitlets, via IAA synthesis, over developmentally delayed fruit, i.e., the primigenic dominance (PD) theory [46]. Our findings do not refute these hormonal regulation hypotheses, but they challenge their broad applicability and suggest that fruit set depends not simply on the number of developed or non-developed seeds within an individual fruit and their capacity to produce IAA, but instead on a fruit’s seed content relative to all other fruits’ seed contents within the population. Several studies have documented a positive relationship between rapid growth rates of apple fruitlets and set [47]. Concomitantly, seeds purportedly contribute to a fruit’s demand for growth resources, such as minerals and carbohydrates, and/or altered hormonal status [48,49]. In the present study, seed content was significantly, positively related to fruit mass, irrespective of cultivar or net treatment (Table 2), corroborating these principles.

The use of nets, in combination with wind pollination, to regulate fruit set and eliminate a reliance on chemical thinning, with all the associated challenges, is novel. Crop load of open-canopy, non-netted control trees in nearly all cultivars and years illustrates the oversetting condition that requires additional steps to reduce excessive crop; this is not only paramount for fruit quality the year of production, but requisite to avoid the economic consequences of biennial bearing. Wind pollination represents a plausible and sustainable strategy in agronomic apple plantations but will require reconsideration of planting designs to facilitate adequate pollen loads to ensure consistent fertilization and yield.

## 4. Materials and Methods

Site and plant material: This experiment was conducted in 2021 and 2022 at the Michigan State University (MSU) Clarksville Research Center (CRC) in Clarksville, Michigan (42°50′27″ N 85°14′36″ W). The planting design comprised three cultivars (‘Gala’, ‘Honeycrisp’, and ‘Fuji’), each planted in five consecutive rows. Row dimensions were 40 m (length) and tree spacing within rows was 0.91 m. All rows (within cultivar and between adjacent cultivars) were separated by a 3.35 m grassed alleyway. No pollinizer cultivars were interplanted within rows. ‘Honeycrisp’ trees were grafted on ‘Geneva^®^ 11’ rootstock, and ‘Fuji’ and ‘Gala’ trees were grafted on ‘Bud 9’ rootstock. Notwithstanding the exclusion of chemical thinning, all trees received standard commercial horticultural and pest management practices throughout the season. The experimental design was a randomized complete block design (RCBD) with five blocks per cultivar; each block comprised three contiguous trees. Two treatments were compared: whole canopies completely enclosed at 0% open bloom (i.e., netting applied between ‘open cluster’ and ‘pink’ phenology stages), using Alt’-carpo nets (9% reduction in PAR, 2.8 mm × 4 mm weave, Helios^®^ anti-hail systems, Bergamo, Italy) and an open-canopy, non-netted control. No insects were observed under the nets during the period of the study. Dates coinciding with the ‘pink’ phenology stage and full bloom were 22 April and 4 May in 2021, and 11 May and 14 May in 2022.

Meteorological data: Wind direction and speed (Figure 4), and temperature and relative humidity (Figure 3) were recorded at 15 min intervals between the phenology stages ‘pink’ and ‘petal fall’ from a calibrated weather station located within 100 m of the trial sites. Data were uploaded to the Michigan Automated Weather Network (MAWN) and Enviro-weather program https://mawn.geo.msu.edu/dod.asp (accessed on 15 June 2023).

Plant measurements: In 2021 and 2022, fruit was calculated for each tree by dividing the number of fruitlets remaining after ‘June drop’ (i.e., the natural fruitlet abscission period occurring ~40 days after bloom) by the number of flowering clusters at bloom (Table 1). After ‘June drop’, 50 fruit were destructively sampled from each replicate of each treatment and cultivar: 25 abscised fruit, collected within the nets of netted trees and from a single piece of net suspended below the open-canopy non-netted trees but above the ground, and 25 retained fruit selected randomly within canopies of each treatment. Each fruit was sectioned, and the total number of developed and non-developed seeds recorded (Table 2). When fruit development reached physiological maturity, the total number of fruit per tree was collected and divided by the cross-sectional area (cm^2^) of the tree’s trunk, calculated from circumference measurements, to generate indices to express fruiting efficiency (Table 1).

DNA extraction and genotyping: In 2021, seeds were extracted from a random selection of fruit (*n* ≥ 25) from each treatment and cultivar at fruit maturity and stored at 4.4 °C for 80 d. All seeds from an individual fruit were sown in the same pot, requiring a total of ~150 pots. The germination rate was determined by the ratio of emerged seedlings to the total number of seeds sown per fruit (Figure 1). Trees were placed in a greenhouse at 21 °C and provided a 16 h photoperiod. After three weeks of growth, young apple leaves were harvested from individual plants and lyophilized over 12 h (Virtis Genesis 25EL freeze dryer (BiopharmaProcess Systems, Winchester, UK)). DNA extraction was performed according to the method outlined by [50]. A pre-extraction purification step using a Qiagen column was implemented to reduce the content of phenolic compounds to enhance genotyping accuracy, as noted by [51]. The extracted DNA was then diluted to an average concentration of 2.5 ng per PCR reaction. The same protocol was applied to five separate cultivars in the orchard from fresh leaves collected after ‘petal fall’: Gala’, Fuji’, and ‘Honeycrisp’ (from the same planting as the net experiment), and ‘Red Delicious’ and ‘Golden Delicious’ from a nearby planting. These samples were intended to comprise potential pollen donors to trees of either treatment.

Genotyping was performed with 16 SNP markers previously developed and converted to Kompetitive Allele Specific Polymorphism (KASP) markers by [50] for distinguishing among apple cultivars (Supplementary S1). The primer was designed and genotyping conducted via the KASP™ system (Ag-Biotech Inc. Monterey, CA, USA), following the protocols described by [50]. PCR amplification was conducted using a touchdown approach with the following thermal cycling conditions: an initial denaturation step at 94 °C for 4 min, followed by 10 cycles consisting of denaturation at 94 °C for 1 min, annealing for 1 min beginning at a temperature of 10 °C above the primer-specific annealing temperature and decreasing by 1 °C per cycle, and extension at 72 °C for 1 min. This was succeeded by 25 cycles of denaturation at 94 °C for 1 min, annealing at the designated annealing temperature for each primer pair for 1 min, and extension at 72 °C for 1 min. The process concluded with a final extension step at 72 °C for 7 min. Analysis revealed that three markers exhibited complete homozygosity when comparing seedling DNA with that of pollinizer cultivars; thus, these markers were excluded from further PCA analysis, leaving 13 markers for subsequent comparisons.

## Figures and Tables

**Table 1:** Fruit set, crop load (number of fruit per cm^2^ of trunk cross-sectional area) and harvested fruit weight of netted and non-netted ‘Gala’, ‘Honeycrisp’, and ‘Fuji’ trees in two years. Data are means of four or five single-tree replicates; *n* = 25 fruit.

	Fruit Set		Fruit Number		Fruit Size	
	(Fruit Number/Cluster)		(Fruit/TCSA)		(g)	
	2021	2022	2021	2022	2021	2022
	‘Gala’					
Non-netted	1.1 ± 0.1	1.8 ± 0.1	17.7 ± 1.5	13.9 ± 1.6	131.5 ± 2.6	171.1 ± 6.4
Netted	0.9 ± 0.1	1.1 ± 0.1	5.2 ± 0.6	8.5 ± 0.8	151.5 ± 5.5	173.4 ± 4.3
*p*	**		***		*	
	‘Honeycrisp’					
Non-netted	1.5 ± 0.2	1.8 ± 0.2	10.1 ± 1.8	10.8 ± 0.9	245.5 ± 9.6	295.9 ± 7.1
Netted	0.5 ± 0.04	0.9 ± 0.06	1.4 ± 0.1	8.5 ± 0.6	311.1 ± 8.1	286.6 ± 13.9
*p*	***		***		*	
	‘Fuji’					
Non-netted	0.3 ± 0.01	1.4 ± 0.1	2.1 ± 0.6	12.1 ± 0.9	250.9 ± 7.1	187 ± 4.6
Netted	0.2 ± 0.07	0.8 ± 0.06	1 ± 0.2	6.8 ± 0.6	248.9 ± 5.7	198.8 ± 7.4
*p*	***		***		0.39	

*, **, *** Significant at *p* < 0.05, 0.01, or 0.001, respectively.

**Table 2:** Relative regression effect of seed number on fruit abscission. Data collected after June drop in netted and non-netted ‘Gala’, ‘Honeycrisp’, and ‘Fuji’ trees. Data means of four or five single-tree replicates; *n* = 25 fruit. Year is a random factor.

Treatment	Fruit Set Status	Seed Number Per Fruit		*p* Abscission
		2021	2022	
		‘Gala’		
Netted	Retained	4.43 ± 0.36 a	8.2 ± 0.5 a ^y^	*** ^z^
	Abscised	2.25 ± 0.26 b	7.5 ± 0.57 a	
Non netted	Retained	1.75 ± 0.37 b	5.2 ± 0.24 b	*
	Abscised	0.34 ± 0.07 c	4.1 ± 0.18 b	
		‘Honeycrisp’		
Netted	Retained	6.02 ± 0.55 a	6.93 ± 0.68 a	***
	Abscised	3.06 ± 0.27 b	4.37 ± 0.77 ab	
Non netted	Retained	1.17 ± 0.48 c	5.09 ± 0.57 a	***
	Abscised	0.12 ± 0.08 c	2.6 ± 0.62 b	
		‘Fuji’		
Netted	Retained	6.35 ± 0.59 a	8.45 ± 0.62 a	***
	Abscised	1.67 ± 1.12 b	3.6 ± 0.81 b	
Non netted	Retained	4 ± 2 ab	5.41 ± 0.72 b	***
	Abscised	2.2 ± 1.46 b	2.6 ± 0.7 b	

^z^ *, *** Significant at *p* < 0.05, 0.01, or 0.001, respectively. ^y^ Mean separation among treatments by Tukey HSD (*p* < 0.05), whereby means associated with different letters are significantly different.

## Data Availability

The raw data supporting the conclusions of this article will be made available by the authors on request.

## Supplementary Materials

The following supporting information can be downloaded at: https://www.mdpi.com/article/10.3390/plants14081196/s1.
